# Valorization of digestates from organic solid waste as fertilizers, soil improvers, and agricultural prebiotics: panorama and perspectives

**DOI:** 10.1007/s13205-025-04507-y

**Published:** 2025-09-10

**Authors:** Ana Laura Zapata-Morales, Ivan Moreno-Andrade

**Affiliations:** Unidad Académica Juriquilla, Instituto de Ingeniería, Universidad Nacional Autónoma de México, Blvd. Juriquilla 3001, C.P. 76230 Querétaro, Qro. Mexico

**Keywords:** Agricultural prebiotics, Digestates, Fertilizers, Organic solid waste, Soil improvers

## Abstract

Digestate, a byproduct of anaerobic digestion, has emerged as a sustainable and viable alternative to chemical fertilizers in agriculture. Several studies have demonstrated that its application can enhance soil microbial biomass, nitrogen mineralization, and nutrient availability without adversely affecting soil structure or microbiological activity. Although the use of digestates is still limited, a global upward trend is emerging worldwide. Promising results, such as increased root growth and improved germination rates, have been reported. However, utilizing digestates without appropriate treatment or quality control may pose risks to human health, soil microbiota, and the environment. The levels of certain contaminants, particularly heavy metals, in digestates can vary significantly. Although in many cases, they are within the limits established by organizations such as the FAO, WHO, and the European Economic Commission, some values may exceed them and pose an environmental risk. From a microbiological perspective, it has been observed that digestates can stimulate beneficial bacterial communities, favoring greater bacterial growth. This review examines the current landscape of the use of digestates derived from organic solid waste, highlighting their potential as fertilizers, soil improvers, and agricultural prebiotics, based on their physicochemical characteristics and their impact on agroecological systems. Nevertheless, their safe use requires strict quality monitoring and post-treatment strategies, particularly in regions such as Latin America, where regulatory frameworks are limited. Establishing robust standards will be key to ensuring their sustainable application in agriculture.

## Introduction

Population growth and industrial expansion have led to various environmental challenges, one of the most significant being the accumulation of organic solid waste (OSW). This waste originates from human activities, including food disposal from households, restaurants, and cafeterias, as well as industrial byproducts from factories and daily operations. Unfortunately, a significant portion of this waste often ends up in landfills and dumpsites, posing severe threats to soil quality, groundwater, and surface water, resulting in environmental degradation and health hazards for local communities. Issues such as respiratory infections, foul odors, and pollution are commonly associated with unmanaged waste in the environment (Dharmendra 2022). According to data from the World Bank, approximately 2.01 billion tons of municipal solid waste are generated each year, with at least 33% of this waste not being safely managed, and it is expected that by 2050, the global waste growth will reach 3.4 billion tons (Kaza et al. 2018). Addressing these issues requires concerted efforts in waste reduction, recycling, and adopting sustainable waste management practices to mitigate the adverse impacts on ecosystems and human health.

Research has focused on finding alternatives for waste treatment, and anaerobic digestion (AD) is emerging as a key technology for the sustainable management of organic waste. AD is a microbial process of biotransformation of organic material in an oxygen-free environment. This dynamic and complex process involves the interaction of different bacterial species, producing biogas and a byproduct known as digestate.

In Latin America, AD is often used to treat manure in dairy and pig farms through covered anaerobic lagoons installed in small- and medium-scale decentralized plants. However, in these facilities, the production of biogas and digestate is rarely adequately monitored due to budgetary and technological limitations in the region. Consequently, biogas and digestate production are also limited (Miramontes-Martínez et al. 2022). One of the most viable options for utilizing digestate is as a biofertilizer, soil improver, and agricultural prebiotic. This review aims to describe the properties and characteristics of digestates from organic solid waste AD treatment, their proposed uses, and applications.

## Organic solid waste

Waste can be defined as any unwanted element or substance discarded by the generator. It can be used, transformed into a new product with economic value, or finally disposed of. Depending on their characteristics and origins, waste is classified into three broad categories: (i) urban or municipal solid waste (MSW), (ii) special handling waste, and (iii) hazardous waste. Currently, the generation of MSW is one of the greatest concerns worldwide. MSW is generated in dwelling houses because of the elimination of materials used in domestic activities, such as consumer products and their containers, packaging, or any other activity that takes place within establishments or on public roads. MSW includes organic solid waste (OSW), such as food waste generated by cafeterias, restaurants, industrial feeders, etc.

One-third of the food generated globally is wasted, amounting to approximately 1.3 billion tons of unconsumed food each year (FAO 2011). This massive amount of waste has led to the need for alternatives in its management. However, managing and disposing of organic solid waste can be very costly, and open-air dumps are currently the most used method for its disposal (Kaza et al. 2018). Unfortunately, the emission of CO_2_ from these dumps was estimated to have reached 1.6 billion tons in 2016, and this number is expected to grow to 2.38 billion tons per year by 2050 if the current disposal method remains unchanged. Organic waste dumped into landfills can release a significant amount of methane gas, which absorbs infrared radiation and contributes to global warming and climate change (Kaza et al. 2018). Besides food waste, sewage sludge, a by-product of biological wastewater treatment plants, is also a major OSW material. There is growing interest in finding alternative ways to use and manage OSW; the AD process is one of the most popular and beneficial methods. This process involves the decomposition of organic waste by a series of microorganisms under free-oxygen conditions, resulting in the production of biogas and an effluent with valuable fertilizing and soil-improving properties (O’Connor et al. 2022; Vasco-Correa et al. 2018).

## Digestates of organic solid waste for soil application

Over the last decade, the use of organic solid waste for biogas production as an alternative biofuel has increased significantly, resulting in the generation of digestate as the final byproduct. This waste has sparked growing interest in agriculture, as both its solid and liquid fractions can be used as a source of nutrients, containing nitrogen (N), phosphorus (P), potassium (K), and micronutrients in plant-assimilable forms (Brychkova et al. 2024). AD is a technology that converts organic waste into biogas through the degradation of organic matter by microorganisms in four stages: hydrolysis, acidogenesis, acetogenesis, and methanogenesis (Vasco-Correa et al. 2018). Hydrolysis involves the breakdown of large organic molecules (lipids, carbohydrates, and proteins) by fermentative bacteria into smaller organic molecules (glucose, fatty acids, and amino acids) (Patel et al. 2017). In the acidogenesis step, the molecules are converted into fatty acids (VFA) (such as acetate, propionate, and butyrate) and byproducts such as CO_2_, H_2_S, NH_3_, and CO_2_ (Zhang et al. 2014). The final stage, methanogenesis, involves the complex interactions between methanogens and enzymes to convert acetate and H_2_ into CH_4_ (Patel et al. 2017). Each stage of AD requires specific conditions and operations for successful development. For example, the acidification stage requires low hydraulic retention time and an acid pH, while methanogenesis is promoted at higher hydraulic retention times and pH values (Pramanik et al. 2019), which are fundamental to determining the quality of biogas and digestate.

Digestate is a mixture of partially degraded organic matter, microbial biomass, and inorganic compounds (Alburquerque et al. 2012). Digestate can be separated into liquid and solid fractions through physical methods. Evidence from the literature suggests that the solid fraction of digestate has a positive impact on all groups of soil microorganisms. In contrast, the liquid fraction is beneficial for bacteria but has negative effects on mycorrhizal and saprophytic fungi (van Midden et al. 2023). For this reason, there has been a growing interest in using digestates in agriculture recently, due to their beneficial properties and potential as a nutrient source (Vaish et al. 2022). They are recognized for their effectiveness as soil improvers, biofertilizers, and agricultural prebiotics because they contain N, P, K, and micronutrients in forms that plants can readily absorb (Brychkova et al. 2024). Furthermore, digestates have been reported to contain a considerable amount of residual organic carbon, as well as humic acids, fulvic acids, carboxylic acids, amino acids, fatty acids, auxins, gibberellins, and other bioactive compounds that can stimulate plant growth (Möller and Müller 2012; O’Connor et al. 2022; Scaglia et al. 2015).

Initial research on the use of digestates focused on their physicochemical characterization, as their agronomic value depends mainly on the characteristics of the raw material or substrate used, the microbial community present, the operating conditions, and the type of AD process employed. This characterization is essential to assess their potential in agriculture. Table 1 presents research aimed at characterizing digestates.

**Table 1:** Examples of characteristics and content of different digestates

Substrate	Country	pH	TS (%)	C (%)	N (%)	P (%)	K (%)	References
OFMSW	Italy	7.9	0.7–51.2	12.8–22.7	1.09	1.49	0.78	Peng and Pivato (2019)
OFMSW	Canada	8.5	–	–	–	1.0**	32**	McLachlan et al. (2002)
OFMSW	Italy	8.3	0.36	0.27	0.08	0.002	–	Pognani et al. (2009)
OFMSW*	USA	7.7	–	41.5	1.03	760**	12,200**	Fernández-Bayo et al. (2017)
Food waste	England	4.4	17.1	-	0.011	0.006	0.004	Rigby and Smith (2013)
Food waste	Italy	8.4–8.9	–	30.2	1.6–3.5	0.5–0.9	0.4–0.5	Grigatti et al. (2019)
Food waste and cow manure	Chile	7.5–8.5	–	15.9	5	< 5	< 5	Vega and Silva (2020)
Food waste, manure, palm oil mill effluent	Nigeria	6.9	21.6	–	3.8	0.11	0.13	Ndubuisi-Nnaji et al. (2020)
Food waste	Brazil	7.8	0.0012	–	0.12	0.049	–	Torres et al. (2018)

*OFMSW* organic fraction of municipal solid waste, *Food Waste* as mixture of bread, cooked meat, fruits, and vegetables

*Considered the process operated as mesophilic condition

**As ppm

One of the first characteristics assessed for the direct application of digestate to soil is pH, as it significantly influences nutrient availability and promotes microbial growth. The optimal pH range for agricultural soils is between 6.5 and 8.0, which indicates slightly alkaline conditions (Möller and Müller 2012). As shown in Table 1, most of the digestates evaluated had a pH range of 6.9–8.9, which is suitable for application in agricultural soils. However, one of the digestates, obtained from food waste as a substrate in the AD process, had a pH of 4.4. This value corresponds to a strongly acidic pH, which can limit the availability of nutrients for crops. This acidity is likely due to both the operating conditions of the process and the heterogeneous mixture of foodstuffs used as a substrate.

One important physicochemical characteristic of digestates is the total solids (TS) content. A higher TS concentration indicates a greater amount of organic matter, which have a positive impact on soil quality. As shown in Table 1, the TS content varies widely, primarily depending on the type of substrate used and the operating conditions of the anaerobic digestion (AD) process. This TS content is closely linked to the carbon content in the digestates. For example, the digestate derived from the organic fraction of municipal solid waste (OFMSW), as reported by Peng and Pivato (2019), shows a TS percentage ranging from 0.7 to 51.2%, with carbon content between 12.8 and 22.7%, confirming that the higher TS concentration correlates with a higher proportion of organic carbon.

The nitrogen concentration in digestate is directly related to the nitrogen content of the substrates used during the AD process. This suggests that protein-rich substrates, such as food waste, manure, and slaughterhouse by-products, tend to produce digestates with higher nitrogen content (Möller and Müller 2012). This correlation is evident in the data presented in Table 1, where the digestate evaluated by Vega and Silva (2020), obtained from food waste and cow Manure, shows a nitrogen percentage of 5%, one of the highest values recorded. Nitrogen in digestates is important because it is an essential element for plants, as it is part of chlorophyll, nucleic acids (DNA and RNA), amino acids, and proteins. Therefore, nitrogen deficiency in plants often manifests as chlorosis (yellowing) and reduced growth (Möller and Müller 2012). Torrisi et al. (2022) reported highly benefited from the administration of liquid digestate in citrus nurseries, increasing the total chlorophyll level in plants (2.97 ± 0.31 mg/L) compared to control (1.90 ± 0.23 mg/L) and mineral fertilizer (1.99 ± 0.25 mg/L), presumably due to the higher ammonium content of the digestate.

Research on the characterization of digestate has noted the presence of trace elements (TE) such as iron (Fe), sulfur (S), manganese (Mn), magnesium (Mg), nickel (Ni), copper (Cu), and zinc (Zn), which are essential in small amounts but potentially toxic in excess, are also present in digestates (Almeida et al. 2019). While these elements are essential in small amounts, they can become toxic at higher concentrations (Almeida et al. 2019). Once applied in soil, TEs interact with the soil matrix through various mechanisms such as adsorption, complexation, and redox reactions, which ultimately determine their mobility and bioavailability. Factors such as soil pH, redox potential, organic matter, and cation exchange capacity play critical roles in modulating these processes (Almeida et al. 2019). Although they may be naturally present in the AD substrate, their concentration largely depends on the origin of the substrate, the type of bioreactor used, and whether mono-digestion or co-digestion is applied (Ezebuiro and Körner 2017).

Trace elements can be externally added as individual compounds or in combinations through nutrient solutions targeting the microorganisms in the digester (Garuti et al. 2018). Table 2 presents various studies that characterize the TE content of different digestates. It is important to note that, for proper crop development, the soil must contain nutrients such as Mg, Zn, Mn, Cl, Cu, and Fe in small amounts. As shown in Table 2, digestates with the highest concentrations of TE primarily come from substrates derived from domestic and agro-industrial waste, attributed to the diversity and mix of waste used. To evaluate the environmental risks and agricultural benefits of digestate application, a comprehensive analysis of TE content is essential, including total concentration, speciation, and fractionation (van Hullebusch et al. 2016).

**Table 2:** Trace element content of different digestates from organic solid waste

Feedstock	Ca (g/kg)	S (g/kg)	Mg (g/kg)	Fe (g/kg)	Mn (g/kg)	Cu (g/kg)	Zn (g/kg)	References
Urban solid waste	8	–	–	14	–	–	–	García-Albacete et al. (2014)
Agroindustry waste	9–65	2.9–14.7	4.1–24.6	0.46–7.9	0.24–1.1	0.014–0.27	0.072–2.2	Monlau et al. (2016)
OFMSW	26.5	5.5	2	3.5	0.135	0.049	0.081	Arab and McCartney (2017)
Household organic waste	47.6	12.2	4.9	26.9	0.278	0.138	0.452	Løes et al. (2018)
Fruit waste	0.0003	0.0027	0.002	0.0038	0.033	0.051	2.46	Serrano et al. (2020)
Sludge and agricultural waste	0.89	–	0.76	1.48	0.51	0.088	0.142	Ezemagu et al. (2021)
Bagasse and agricultural waste	1.07	2.30	2.21	0.17	0.04	0.01	0.05	Morquecho (2020)
Food waste and garden waste	380 mg/L	250 mg/L	100 mg/L	6	–	4	2	Santos et al. (2023)

In digestates, calcium (Ca) is one of the most abundant elements, with concentrations reaching up to 47.6 g/kg in digestates from domestic waste and ranging from 9 to 65 g/kg in agro-industrial origin. Ca is essential for plant growth and development, as it participates in signaling, metabolism, and cell growth processes (Weng et al. 2022). Its deficiency leads to cell death in the apical meristems (Ren et al. 2021). Fe and Mg also play a fundamental role in plant physiology and soil health. Iron aids in the stabilization of organic carbon in the soil, while magnesium is critical for several metabolic processes, being a key component of chlorophyll and acting as an enzyme activator (De Sousa Ferreira et al. 2023).

Although these elements are essential for plants and soil microorganisms, they can be toxic at high concentrations. Their impact will depend on the origin and treatment of the digestate, as well as soil characteristics and crop conditions, including the land's agricultural history, previous soil treatment, and irrigation practices. Over time, repeated application of digestates may lead to TE accumulation in the soil, potentially affecting soil health, microbial activity, and plant uptake pathways (Almeida et al. 2019). While some studies suggest that mineral phases in biosolids can immobilize metals and reduce environmental risk (Hettiarachchi et al. 2006), other authors propose that long-term mineralization of organic matter results in the gradual release of bound TEs, increasing their mobility and bioavailability (McBride 1995).

These opposing perspectives highlight the necessity for comprehensive long-term evaluations of digestate use, including field-based monitoring of TE speciation and fractionation to accurately assess the environmental fate of metals introduced through digestates (Almeida et al. 2019). These considerations are crucial when determining the appropriate dosage of digestate to apply (Almeida et al. 2019), as well as its potential as a biofertilizer, soil enhancer, or agricultural prebiotic. Furthermore, both field and lab studies are necessary to understand the long-term impacts on soil fertility, TE uptake by plants, and potential entry into the food chain. Such evaluations are vital for promoting the use of digestates within sustainable agricultural frameworks.

## Use of digestates as fertilizers, soil improvers and agricultural prebiotics

Soil is a natural and dynamic component of the Earth’s crust, composed of layers known as horizons that contain mineral materials, organic matter, water, and air, which support the growth of plant roots (Bandick and Dick 1999). However, soil quality can be negatively impacted by machinery, fertilizers, pesticides, agrochemicals, organic amendments, and the type of crops planted. Improving soil quality is essential for agriculture and plays a crucial role in food production. Therefore, it is important to identify alternatives that can improve soil quality while minimizing environmental impact. One such alternative is the use of digestates, which can serve as biofertilizers, soil improvers, and prebiotics (van Midden et al. 2023; Palansooriya et al. 2023; Yadav and Yadav 2024).

### Potential of digestates as biofertilizer

Biofertilizers are products containing nutrients and microorganisms that promote crop growth and development by increasing the nutrient supply, encouraging the growth of beneficial microorganisms, and improving the plant's nutrient absorption capacity (Vessey 2003). They are a natural and effective alternative to chemical fertilizers, with advantages such as lower cost and eco-friendliness (van Midden et al. 2023). Crops require small amounts of TE, which can have a biostimulant effect and play a role in plant enzyme systems. However, the characterization, quantification, and understanding of the effects of biostimulant compounds from biofertilizers on plant growth are still in the early stages of research. Many studies have focused on hydroponic systems rather than soil (Antón-Herrero et al. 2021; Barone et al. 2019). Some studies have identified the promotion of beneficial arbuscular mycorrhizal fungi, and bacteria such as *Bacillus* sp., *Bacillus siamensis*, *Pseudomonas* and *Rhodococcus* promoting plant growth (Pagliaccia et al. 2020; Pastor-Bueis et al. 2017; Qi et al. 2017; Ren et al. 2020)*.*

Several studies have demonstrated that the physicochemical composition of digestates is highly variable, primarily influenced by the type of feedstock used in AD (Table 3), the operational parameters, and any post-treatment processes. Comparative analyses of digestates derived from food waste, manure, agro-industrial residues, and municipal waste reveal substantial differences in nutrient content and phytotoxic effects. For instance, Song et al. (2021) reported that digestates from food waste, while rich in nutrients, exhibited elevated sodium and ammonium levels that induced salinity stress, limiting their agricultural utility unless diluted. Optimal plant growth in spinach, lettuce, and cabbage was observed at 20–40% dilutions, while higher concentrations impeded germination due to oxygen deficiency in the rhizosphere. Conversely, Panuccio et al. (2016) investigated a digestate from Manure, whey, and corn residues, applying a phase separation pretreatment. This approach enabled targeted nutrient analysis, revealing that the solid fraction contained higher levels of K, P, and Ca. Both liquid and solid fractions, tested at various dilutions, demonstrated that concentrations above 50% negatively impacted germination, although the severity of this impact varied by crop, with cucumber showing greater tolerance. These findings underscore the importance of tailoring digestate application strategies based on its composition, the crop species, and the benefits of pretreatment, such as phase separation, to mitigate potential phytotoxicity.

**Table 3:** Examples of digestates from different feedstock as biofertilizers and soil amendments

Digestate from different feedstock	Anaerobic digestion operation	Digestate treatment used	Identified nutrients (mg/L)	Experimental conditions for plant growth	Observations	References
Food waste	40 kg volatile solids (VS) per week, with a production of 0.36 L_methane_/gVS fed	No treatment	NH_4_ (4700), NO_2_^−^ (8.64), Mg (6460), Cl (2853), Ni (68)	**Plant:** Chinese spinach (*A. tricolor*), Chinese cabbage (*B. rapa*), Water spinach (*I. aquatica*), and lettuce (*L. sativa*) **Digestate:** Semi-liquid, diluted to 20%, 40%, 60% and 80% with tap water (v/v)	Plants grown increase by using digestate (20–40%). No growth in 80–100% due to high salinity, high ammonia concentration or low oxygen level in the root zone of the plants	Song et al. (2021)
Manure, milk serum, and maize silage	Process temperature 40 °C, pH 8.0, Digester volume 7420 m^3^, 120 m^3^ loaded per day	Separation liquid and solid fraction	**Liquid fraction:** K (480), P (290), Ca (600), Mg (9), NH_4_ (340), NO_3_ (140) **Solid fraction:** K (960), P (560), Ca (900), Mg (100), NH_4_ (30), NO_3_ (1500)	**Plant:** watercress (*N. officinale*, lettuce (*L. sativa*), and cucumber (*C. sativus*) **Digestate:** Liquid: diluted 0, 10, 25, 50, 100%, and solid: extracted in water (1:5 w/v, 24 h) and then diluted with distilled water (0–100%)	The germination of the seeds and their development is inhibited with the dilutions of 50 and 100% in both fractions of digestates, except with the cucumber seeds	Panuccio et al. (2016)
Cow and pig manure	Digestates from biogas production in a university facility	No treatment	N (5.0), P (73.2), K (810.0), Ca (159.0) Mg (147.5), Fe (4.0), Zn (1.6)	**Plant:** Onion (*A. cepa*) **Digestate:** Semi-liquid, diluted: 20, 35, 50, and 60% with water (v/v)	50% dilution showed the highest crop yield, in growth and height. The higher dilution values do not present significant yields	Coaguila et al. (2019)
OFMSW	Thermophilic digestion at 55 °C, with shaking 60 rpm	Acidification with H_2_SO_4_, up to pH 6.5	N (5.3 g/kg), P (0.7 g/kg), K (3.5 g/kg), Ca (0.52 g/kg), Mg (0.83 g/kg)	**Plant:** Lettuce (*L. sativa*), chard (*B. vulgaris* var. cicla) and spinach (*S. oleracea*) **Digestate:** Solid, doses 0.1/kg of soil	Germination tests in pots. Digestate showed greater germination than soil treated with compost	Salcedo-Serrano et al. (2022)
Flower waste	AD in 2 L sealed glass bottles	No treatment	All in mg/kg: P (111), Na (1011), K (1122), Ca (140) Fe (966), Mn (247), Cu (37.8)	**Plants:** Eggplant (*S. melongena*) **Digestate:** Pots with 25% digestate with 75% soil, 50% digestate with 50% soil, 75% digestate and 25% soil and 100% digestate	No inhibition was observed. Improvements were noted in the roots, shoots, and leaves compared to the control group	Vaish et al. (2022)
Fruit and vegetable wastes	Continuous stirred tank reactor (37 °C), HRT 21 d	No treatment	P (102), K (119), S (72.8), Ca (215), Fe (134), Cu (0.69), Na (881)	**Plants:** Maize (*Zea mays* L.) **Digestate:** 152.3 and 120.5 mL according to the C/N ratio	No significant differences found among any of the treatments when compared to the control	Álvarez-Alonso et al. (2022)
Mixture of cafeteria waste	3785-L plug flow anaerobic digesters Fed with 7% solids 3 times/week per week	No treatment	Mg (4000), S (2000) Fe (15.5), Cu (0.113), Mn (0.743)	**Plants:** Cucumber (*C. sativus*), Lettuce (*L. sativa*) **Digestate:** NKP ratio 2.9: 3.5:0.3% w/w	Cucumber germination was significantly higher with digestate compared to both lettuce and the control group (which used water) Crop height and fruit production increased with increasing rates of digestate application	Lee et al. (2021)

Using digestate from food residues as a fertilizer has been shown to increase the content of macro and microelements in soil and plants (Chiew et al. 2015). However, some studies have also reported the possibility of a negative impact on the soil due to phytotoxicity when using digestate as a biofertilizer (Odlare et al. 2008) and productivity decrease of some crops biofertilizers (Khan et al. 2023), related to inhibitory concentration of some compounds, making necessary a previous evaluation of digestate doses for the increase of the crops production avoiding inhibition of growth.

Empirical evidence supports the use of digestates, particularly those derived from pig slurry, as effective biofertilizers that enhance soil fertility, crop quality, and resistance to both biotic and abiotic stressors (Kouřimská et al. 2009). For example, Coaguila et al. (2019) assessed an untreated digestate from cow and pig manure, which exhibited moderate levels of K (810 mg/L), P (73 mg/L), and N (5 mg/L). In onion crops, a 50% dilution significantly improved plant height and biomass, suggesting that digestates with a balanced nutrient profile can be applied effectively without requiring pretreatment, if concentration is carefully managed.

It was reported that digestates from the organic fraction of municipal solid waste (OFMSW), sewage sludge, and flower waste significantly improved soil nutrient availability and enhanced the growth, biochemical, and yield parameters of *Solanum melongena* (Vaish et al. 2022). Moderate doses of digestates increased the content of chlorophyll, carotenoids, and protein, while maintaining metal concentrations within safe limits. Higher doses (> 75%) induced mild oxidative stress, mitigated by elevated antioxidant activity. Yield improvements of up to 173% confirm the agronomic viability of using digestates. Sica and Magid (2024) investigated the use of a digestate produced from OFMSW (acidified with H_2_SO_4_ to a pH of 6.5) applied in solid form (0.1 kg/kg soil), observing an enhancement in seed germination for lettuce, chard, and spinach, surpassing the performance of conventional compost. The acidification likely improved nutrient bioavailability while mitigating ammonium toxicity, reducing the need for dilution. Collectively, these findings highlight that multiple variables, including substrate origin, treatment strategies, application rate, and crop type, influence the agronomic efficacy of digestates. Therefore, thorough characterization of the digestate is critical to optimize its agricultural use and avoid environmental or phytotoxic risks (Vaish et al. 2022).

### Use of digestates as soil amendments

In recent years, the overuse of chemical fertilizers has led to a marked decline in soil fertility and crop productivity. As a sustainable alternative, the application of soil enhancers has emerged as a promising strategy to improve the physical, chemical, and biological attributes of soils (Elumalai et al. 2025). These amendments promote better aeration, enhance water retention, and increase nutrient availability for plant uptake. A key indicator of improved soil function is the cation exchange capacity (CEC), which reflects the soil’s ability to retain and supply essential cations such as Ca, Mg, K, and NH₄. In this context, digestates have been identified as potential soil enhancers due to their rich content of organic matter and essential nutrients. Beyond supplying nitrogen and phosphorus, digestates stimulate soil microbial activity. Long-term field studies have demonstrated that repeated applications of digestate-based amendments can significantly increase microbial biomass and elevate soil N and P levels, leading to enhanced soil fertility. Almeida et al. (2019) reported an 11% increase in substrate-induced respiration following digestate application, suggesting an elevated microbial capacity to mineralize organic matter and thereby contributing to sustained soil health and productivity.

Odlare et al. (2008) conducted a 4-year study in Sweden on a soil that had not been fertilized for over 20 years and planted cereals. They found that the chemical properties of the soil did not change significantly in the short term when modified with organic waste, including digestates. However, compared to other treatments such as pig manure, cow manure, compost, and inorganic fertilizer, soils treated with liquid digestate from domestic waste showed the highest increase in microbial biomass, nitrogen mineralization rate, and potential oxidation of ammonia. In another study, two types of soil were supplied with different types of materials to understand their role as soil improvers (wine waste digestates, highly stabilized and poorly stabilized compost). The study found that anaerobic digestates from the wine industry mineralized nitrogen at a higher rate than their counterparts (Canali et al. 2011).

The use of dry topsoil (Hanford sandy loam) with two different solid digestates has been studied (Fernández-Bayo et al. 2017), specifically mixing organic waste digestate (comprising food, agricultural, and green waste) with another containing animal feed and green waste. The experiment involved mesocosms with soil mixtures, where the dry soil was modified with one of the two digestates to achieve a 1.5% charge (based on dry weight). Additionally, *Brassica nigra* (black mustard) and *Solanum nigrum* seeds (nightshade) were added to a depth of 15 cm within the soil mixtures, observing a positive effect on nutrient availability (P and K) and amendment properties, such as total C content and degree of humification, without inhibition in weed growth resulting from the application of digestates. The study found that biosolarization with digestates did not negatively affect soil properties or humification rates. Additionally, the microbial activity stimulated by these amendments was not sufficient to induce biological soil heating. However, soil treated with mixed waste digestate exhibited a beneficial interaction with solar heating. This suggests that the digestates not only enhance nutrient availability but also potentially contribute to the control of soil pathogens such as harmful nematodes, fungi, bacteria, and insects. Despite these promising findings, it is evident that there remains a scarcity of studies focused on identifying the precise role of digestates in these processes. Further research in this area is warranted to understand better and harness the potential benefits of digestates in sustainable soil management practices.

### Digestates as prebiotics

Prebiotics are a type of biostimulant, generally of natural origin, that include humic and fulvic acids, protein hydrolysates (from plant or animal sources), seaweed and algae extracts, chitosan and other biopolymers, as well as inorganic and mineral compounds such as iron, manganese, and zinc (Alahmad et al. 2023). These substances are applied to plants or the rhizosphere to stimulate natural processes, improve nutrient uptake and nutritional efficiency, enhance tolerance to abiotic stress, and improve crop quality. Although their application is relatively recent, growing evidence supports their effectiveness in promoting plant growth (Alahmad et al. 2023).

Studies on the prebiotic effects on plants and the rhizosphere have included microbial characterization, yield, and growth studies in plants (Yakhin et al. 2017). Some of the microorganisms that have demonstrated prebiotic capacity include mycorrhizal fungi such as *Glomus fasciculatum*, fungi like *Trichoderma viride*, and bacteria like *Bacillus coagulans* and *Pseudomonas fluorescens*, among many others (Alori and Babalola 2018).

**Fig. 1:**
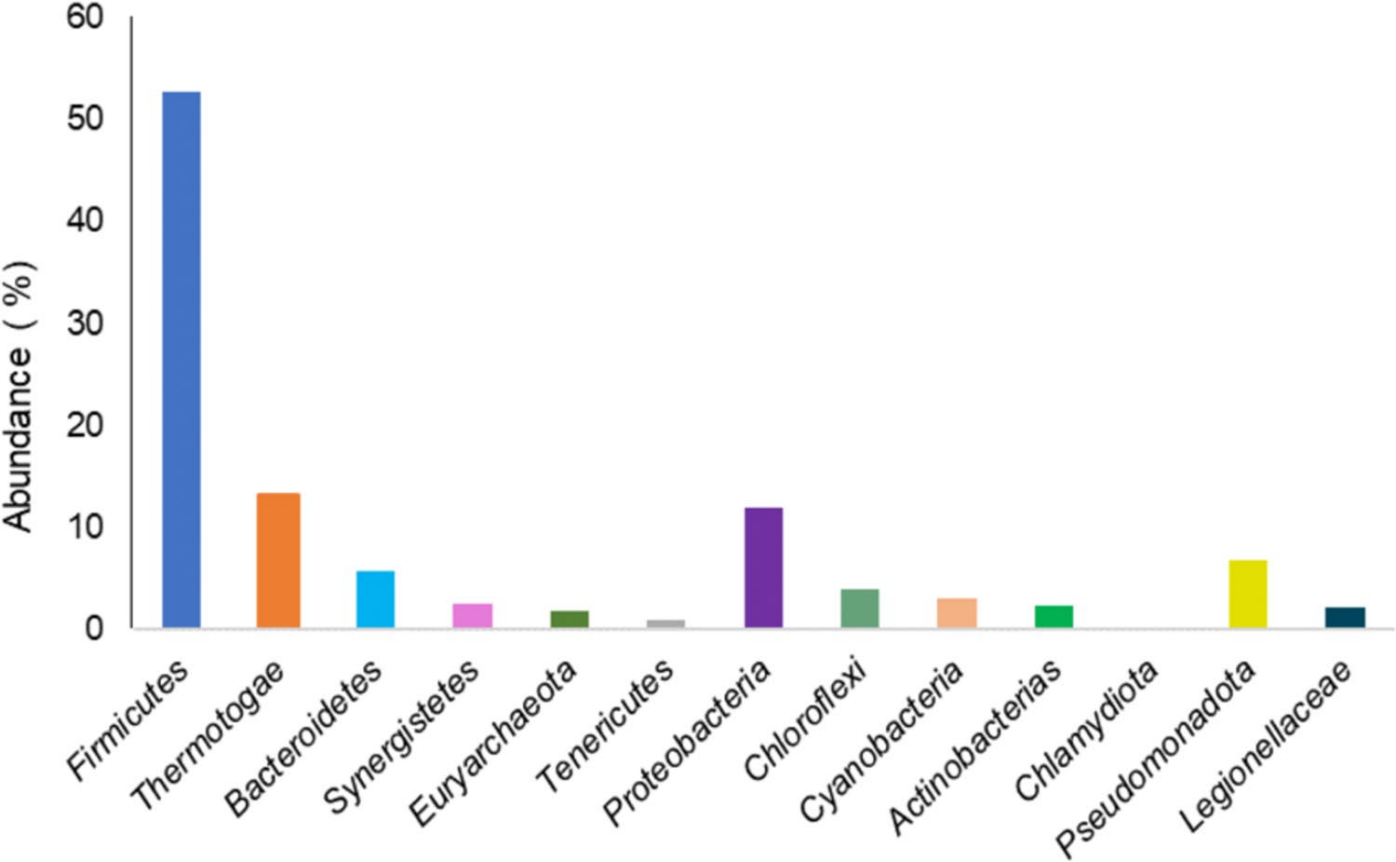
Relative abundance of the main microbial phyla in digestates reported in the literature (Pastor-Bueis et al. 2017; Fernández-Bayo et al. 2017; Fernandes et al. 2020; Pagliaccia et al. 2020; Song et al. 2021)

There is a lack of research on the microbiological properties of digestates and their potential role as biostimulants in soil ecosystems. Further investigation is needed to determine how digestates interact with soil microbiota and their impact on soil health. By exploring microbial dynamics and evaluating their efficacy as biostimulants, innovative approaches in sustainable agriculture and soil management can be developed. Figure 1 shows the microorganisms most commonly identified in digestates. Firmicutes and Proteobacteria are the most abundant phyla in digestates, while the *Pseudomonas* and *Bacillus* genera are the most representative in terms of microbial abundance, exhibiting plant growth-promoting capacity.

Table 4 presents the studies that have been conducted to explore the potential of digestates as agricultural prebiotics, highlighting how their composition and functionality vary depending on the original substrate and the conditions of the AD process. C/N ratios in digestates can lead to differences in microbial growth. Fernández-Bayo et al. (2017) showed that lower C/N ratios increase genes related to the degradation of hemicellulose and lignin, indicating a greater potential for the transformation of organic waste. This suggests that adjusting the C/N can modulate the functional profile of the digestate microbiome, enhancing its potential as a soil amendment. A study conducted by Manfredini et al. (2021) demonstrated that a high concentration of dissolved organic carbon (above standard field application doses) increased microbial activity and abundance for several weeks, in agreement with Fernandes et al. (2020).

**Table 4:** Studies of digestates for use as agricultural prebiotics

Digestate substrate origin	Microbiological characterization	Experimentation	Measured parameters	Observations	References
Mixture of green waste (grass and clippings) and dog food (22% protein, 14% fat, 50% carbohydrates and 13% crude fiber)	Abundance in percentage: *Firmicutes* (> 65%) *Thermotogae* (7–25%) *Bacteroidetes* (< 5%) *Synergistetes* (< 5%) *Euryarchaeota* (< 5%) *Tenericutes* (< 5%)	Effect of C/N ratio (17, 20, 23, 27, and 34) on the microbiome of the AD digestates. No experimentation with soil	Bacterial abundance, genes encoding proteins associated with hemicellulose, lignocellulose, lignin and cellulose degradation	The C/N ratio affects the digestates microbiome. A ratio > 27 enriches the Clostridiales family. C/N ratio < 23 generates solid digestates rich in genes encoding proteins with the capacity to protect cells from N intermediates, accumulation of rhamnosides and increases maltose and galactomannans degradation	Fernández-Bayo et al. (2017)
Organic waste	Not identified genera, only relative abundance of bacterial 16S rRNA gene	Set up in 300 g of soil. Digestate application rate of 10 g (dry weight) per 100 g of soil (equivalent to a field application of 100 mg/ha)	Microbial activities: dehydrogenase, arylsulfatase, b-glucosidase, b-glycosaminidase, alkaline phosphatase and protease	16S rRNA gene relative abundance in soil with digestate showed an improvement in gene copies at all treatment times (highest at 60 days). Increasing nutrients increases dehydrogenase activity at 60 days, and glucosidase is unchanged	García-Albacete et al. (2014)
Mixture of food waste and beer mash (a mixture of water and malted grain)	*Pseudomonas* sp. *strain HAL1, Pseudomonas moorei strain 28, Acinetobacter* sp.* KO14-1, Glutamicibacter soli strain cjy151, Pseudomonas* sp.* strain Di5*	Growth of *Citrus sinensis* tested in a mix of silica sand (33%), redwood shavings (33%), and coir (33%). Application of liquid digestate by drip irrigation system (7.5 L/h). The excess nutrient solution from each pot was gravity-drained into a reservoir and recirculated	Effect of digestate on leaves, roots, rhizosphere, and bacterial populations Increase of N and C content in the soil, siderophore production, and iron acquisition	Beer Mash (BM) digestate increased average N by 166% and C by 164% while food waste (FW) digestate increased N by 152% and C by 259%. Bioinformatics analysis indicated that all bacteria of BM and FW have genes involved in the production of siderophores and iron acquisition, participate in the ABC output system and TonB family proteins, some of these bacteria also possess genes involved phosphate solubilization and nutrient facilitation (Acetolactate synthase, PadR transcriptional regulator)	Pagliaccia et al. (2020)
Buttermilk	*P. kudriavzevii* *L. fermentum* *L. helvéticus* *L.hilgardii* *L. rhamnosus* *L. zeae*	Digestates from AD buttermilk serum previously pasteurized and inoculated with a Culture at 2% v/v of *L. rhamnosus* Separation of biostimulants	Microbiological characterization and capacity of lactic acid and protein hydrolyzation (as soil biostimulant)	The use of digestates increased 4.73% the microorganisms as probiotics (10^11^ CFU/g), 13.54% hydrolyzed proteins, 62.07% lactic acid, and 3.55% minerals	Caballero et al. (2020)

Garcia-Sanchez et al. (2015) evaluated the effect of an organic waste digestate directly on the soil, applying 10 g of digestate per 100 g of dry soil (equivalent to 100 t/ha), in this case, significant increases in the activity of microbial enzymes were observed, especially dehydrogenase at 60 days, reflecting an increase in the biological activity of the soil. An increase in the relative abundance of the 16S rRNA gene was also noted, suggesting that digestate stimulates soil microbial diversity and abundance over time, without significantly altering the activity of enzymes such as glucosidase.

Pagliaccia et al. (2020) analyzed digestates from mixtures of food waste and beer mash, applying liquid digestates to a drip irrigation system to evaluate their effect on *Citrus sinensis*. Increases in soil nitrogen (up to 166%) and carbon (up to 259%) were reported. Furthermore, through bioinformatics analysis, it was identified that the bacteria present possessed genes related to the production of siderophores, iron acquisition, and phosphate solubilization, key functions that promote plant growth and improve nutrient availability.

The application of the liquid fraction and the unseparated digestate (liquid and solid phases) to soil rapidly stimulates microbial activity (Risberg et al. 2017). However, these changes in microbial activity, abundance, and biomass are temporary and often disappear within days after application. This has been observed especially when using the liquid fraction, because the liquid digestate does not provide enough available carbon for soil microorganisms to grow sustainably and is not detectable after a few weeks (Galvez et al. 2012; Iocoli et al. 2019; Barduca et al. 2020; van Midden et al. 2023). In contrast, the application of solid digestate leads to sustained increases in microbial biomass and activity (de la Fuente et al. 2013; Badagliacca et al. 2020; Cattin et al. 2021), indicating that the solid fraction provides a more stable carbon source.

Digestates obtained from pasteurized buttermilk were demonstrated to enhance the growth of lactic acid-producing bacteria (*Lactobacillus rhamnosus*), thereby increasing protein hydrolysis (Table 4). This type of digestate represents a more targeted approach toward the production of agricultural probiotics or products with specific functions in the rhizosphere (Caballero et al. 2020). In this study, biostimulant products, including lactic acid, peptides, and amino acids, along with biomass of *L. rhamnosus,* were purified and evaluated for their soil biostimulant and biocontrol capacities. The presence of lactic acid was found to lead to changes in microbial biodiversity, favoring bacterial genera known to promote plant growth. Additionally, *L. rhamnosus* exhibited biocontrol activity against certain phytopathogenic microorganisms. Using liquid digestates in irrigation resulted in a reduction in pathogenic bacterial diversity and the selective growth of beneficial microorganisms, such as *Pseudomonas putida* (Pagliaccia et al. 2020). The addition of digestate can enrich the medium with nutrients and organic compounds, favoring microbial development, especially when combined with a carbon- and nitrogen-rich source. These results are consistent with those reported by Tiempo (2024), who noted that an increase in carbon availability directly contributes to the increase in microbial biomass.

In specific scenarios, to achieve the best results from digestates for the prebiotic process in soil, it may be necessary to supplement with additional nutrients. Recent research conducted by Holatko et al. (2021) has demonstrated that incorporating supplements, such as humic acid, can significantly enhance the prebiotic activity of digestates. Combining digestates with biocarbon, humic acids, or both can result in a synergistic effect that enhances enzymatic activity and contributes to improving soil properties, including nutrient assimilation by plants (Holatko et al. 2021).

## Impact of digestate characteristics on soil quality

The use of digestates in the soil can promote plant growth by providing essential nutrients such as nitrogen and phosphorus (Cheong et al. 2020). A study by Mickan et al. (2022) demonstrated that incorporating digestate into tomato (*Solanum lycopersicum*) crops promoted shoot and root growth, resulting in increased crop biomass. However, concerns exist regarding the direct addition of digestate to soil, which can lead to nitrogen loss and air pollution due to its high ammonium-nitrogen content (Manu et al. 2021). It has been suggested that the application of liquid fraction and non-separated fraction of digestate to soils rapidly stimulates microbial activity (Meng et al. 2022). Although an initial increase in microbial biomass is observed shortly after digestate application, some studies indicate that these changes in microbial activity and abundance are temporary, diminishing within a few days after application. This phenomenon may be attributed to the selection of microbial communities that are best adapted to the soil’s prevailing conditions.

In various studies, digestates have been found to contain NH_4_ content ranging from 30 to 500 mg/L. This high ammonium content, in combination with the moisture present in digestates, creates favorable soil conditions for different bacterial groups. As nitrogen transforms, the abundance of nitrifying and denitrifying bacterial groups tends to increase (Ogbonna et al. 2018). This phenomenon highlights the importance of understanding the impact of digestate characteristics on soil microbial communities, and nutrient cycling processes. The direct and repeated application of digestates can bring about changes in the physicochemical properties of soil, the full extent of which is still largely unknown. One crucial characteristic to consider is the pH of the soil, which affects the abundance and microbial diversity, as well as the solubility of inorganic and organic compounds like nutrients and metals. Heavy metals have garnered attention due to their potential adverse effects on living organisms and the environment. Studies suggest that applying digestates to soil may lead to the accumulation of heavy metals in both soil and crops. Digestates derived from wastewater, industrial, and urban waste have been found to contain elevated levels of metals, including copper, cadmium, nickel, lead, and zinc, surpassing the established limits deemed acceptable for soil application according to regulatory standards (Coelho et al. 2018). These findings emphasize the importance of conducting thorough assessments and monitoring to mitigate the potential risks associated with heavy metal accumulation resulting from the application of digestate on soils.

The presence of high concentrations of heavy metals in soil can lead to a reduction in enzymes and alter the microbial composition. Although many studies have analyzed the metal content and found it to be below the recommended threshold levels established by law, which makes them safe for use, there are still doubts about the cumulative effects in the long term, especially if the application of the digestates is repetitive. It is important to note that the use of this effluent has been demonstrated to increase production and crop yields, while also contributing nutrients to the soil and enhancing its quality by stimulating the activity of microorganisms.

## Digestates metabolites as fungicides, nematicides, and growth promoters

The use of digestates in agricultural fields for the benefit of soil and crops is a practice that has currently been established due to their characteristics, such as their nutrient content, including nitrogen, phosphates, and potassium, among others. Recently, several studies have reported the presence of metabolites in digestates, which have been observed to exhibit antifungal, bactericidal, nematicidal, and metabolic activity-stimulating properties, among others (van Midden et al. 2023; Oldani et al. 2023). Within this context, some studies extracted different metabolites, generally VFA, and reported stimulating or inhibitory activities in the development of crops (van Midden et al. 2023). Oldani et al. (2023) indicated that the use of digestate from agricultural, municipal, and industrial waste presents nematicidal activity against *Meloidogyne incognita*, one of the root-knot nematodes that directly affects more than 1700 vascular plants and is among the five main plant pathogens (Jones et al. 2013). In this study, it was observed that the 5% and 10% treatments in cucumber crops resulted in a 10% decrease in gall development compared to the control. Likewise, it was observed that the application of digestate did not show phytotoxic effects on cucumber and tomato plants. On the contrary, taller shoots were observed in the pots treated with the 5% and 10% concentrations.

Laboratory experiments have shown that soils treated with digestate exhibit significant reductions in root-knot nematode populations and decreased egg production by cyst nematodes (Das et al. 2022; Xiao et al. 2007), compared to untreated soils. The nematode-suppressive effects of digestates are attributed to several mechanisms including stimulation of antagonistic bacterial communities, the presence of plant-derived nematicidal compounds in digestate mixtures, and elevated concentrations of ammonium and organic acids generated during AD process (Wang et al. 2019; Westphal et al. 2016; Min et al. 2007).

Samaniego and Pedroza-Sandoval (2013) state that VFAs such as acetic acid and propionic acid exhibit properties against phytopathogenic organisms in the soil (concentrations > 307 mg/L). Likewise, Voelkner et al. (2015) have indicated that these organisms die in just minutes because the VFAs modify the osmotic gradient of the cellular membrane of these microorganisms. This effect was evident in their study, where microbiological characterization revealed the absence of fecal coliforms and *Salmonella* spp. in the digestates.

**Fig. 2:**
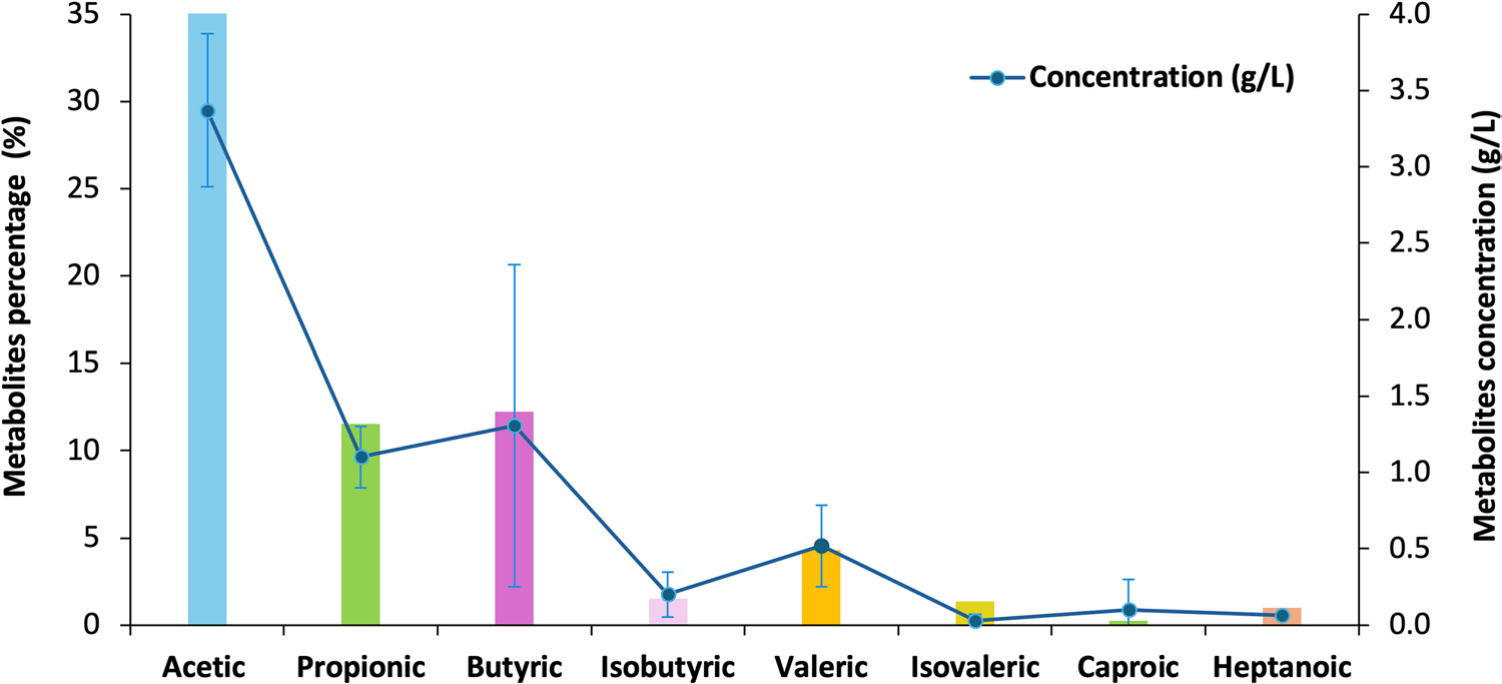
Concentration and percentage of the main metabolites identified in digestates, according to an analysis of data collected in different studies (Oldani et al. 2023; Ghidotti et al. 2018; Huang et al. 2015)

Recent studies aimed at characterizing the metabolites present in digestates have primarily identified VFA (Fig. 2). However, research on the properties of these metabolites is still limited. The most identified metabolite is acetic acid, which accounts for an average of 34% of the digestate composition, followed by unidentified metabolites at 7%, and then propionic acid at 5.8%. Studies have indicated that these compounds have an important role in the health of the soil and crops, according to the study reported by Ramsdale (2008), it was indicated that applying for 15 min and a concentration of 300 mmol/L of acetic acid, more than 95% of *C. albicans* (fungi, opportunist pathogen) dies, likewise it was observed in the study of Samaniego-Gaxiola and Balagurusamy (2013), that when applying acetic, butyric, formic and propionic acid in a concentration of 38 µg/L inhibits the *Phymatotrichopsis omnivore*. This soil-borne ascomycete attacks thousands of plant species and is the causal agent of the disease known as “Texas Rot” (Samaniego-Gaxiola 2007). The above-mentioned emphasizes that digestates are nutrient-rich for the soil and plants. They can also provide metabolites that support crop growth by inhibiting the development of disease-causing microorganisms in crops. It is important to note that further studies are necessary to determine the concentrations of these compounds that plants and soil can tolerate without experiencing negative effects.

## Framework for the use of digestates

Digestate is an emerging by-product of anaerobic digestion, increasingly considered as a nutrient source for agricultural soils. In the European Union, approximately 95% of digestate is applied to farmland (Dahlin et al. 2015). While its use has shown promise, including potential alterations to the soil microbiota, current evidence remains insufficient to characterize its behavior and long-term impact fully. Key research gaps persist regarding digestate stability, particularly in understanding the decomposition processes it undergoes after application, such as organic matter mineralization, nitrogen availability, and mineralization–immobilization dynamics. These processes are critical to understanding the role of digestates in soil nutrient cycling and fertility. Although studies have demonstrated that both liquid and solid digestates can perform as effectively as, or even better than, mineral fertilizers and untreated manure (Nkoa 2014; Chantigny et al. 2010), concerns remain regarding their environmental implications. Risks associated with land application include atmospheric emissions of ammonia and nitrous oxide, nutrient leaching, and contamination through phytotoxic substances, heavy metals, or pathogenic organisms. These findings underscore the need for more comprehensive research to maximize the agronomic benefits of digestate while minimizing environmental risks.

The liquid fraction of digestates retains the majority of N and K, while the solid fraction contains a higher proportion of residual fibers and P. This compositional heterogeneity presents challenges for direct land application, as improper use can lead to nutrient imbalances or environmental risks. Consequently, the implementation of pretreatment processes is strongly recommended before field application. Moreover, regulatory frameworks governing digestate quality and usage remain under development in several countries, including Spain, France, and the United States. In Latin American countries, there is currently no official standard defining permissible limits or guidelines for digestate utilization, underscoring the need for regulatory advancement to ensure the safe and practical application of digestate in agriculture.

In countries where the AD process is used to treat OSW for biogas production, a standardized approach has been established, resulting in the installation of several treatment plants. A proposal has been put forward to treat the digestate produced during this process. The goal of this treatment is to achieve the necessary physicochemical characteristics for its application to soil and to maximize the utilization of its nutrients (Raboni and Urbini 2014). The methods for digestate treatment may include acidification, concentration, separation of solid and liquid fractions, flocculation, and composting, among others (Zamanzadeh et al. 2016).

It is important to control the concentration of heavy metals in the digestate, as well as the presence of pathogens such as *Salmonella* spp., fecal coliforms, and helminth eggs (Raven and Gregersen 2007). This is especially important even when the digestion is thermophilic. Table 5 provides general data on the maximum permissible levels of metals in compost and organic amendments for soil application across various countries. Before applying digestate as a biofertilizer or soil enhancer, it is critical to perform comprehensive soil analyses to assess existing nutrient levels and minimize the risk of nutrient toxicity or soil saturation. Regional variability in regulatory thresholds, particularly for heavy metals, must be considered to ensure safe use. In Latin American countries, where the use of digestate from wastewater treatment plants is becoming increasingly common, access to detailed data on soil and digestate composition is essential for sustainable agricultural applications. Additionally, environmental risks such as groundwater contamination by trace metals must be carefully evaluated, especially in permeable sandy soils (Liu 2016). For instance, long-term application of biogas residues has been shown to introduce measurable quantities of heavy metals (Odlare et al. 2008).

**Table 5:** Maximum permissible levels of trace metals in soil by country (Modified from Nunes et al. 2021; Epelde et al. 2018; Al Seadi et al. 2013; Fekri and Kaveh 2013)

Country	Cd (mg/kg)	Pb (mg/kg)	Hg (mg/kg)	Ni (mg/kg)	Zn (mg/kg)	Cu (mg/kg)	Cr (mg/kg)
Denmark	0.8	120	0.8	30	4000	1000	100
Norway	2	80	3	50	800	650	100
Sweden	1	100	1	50	800	600	100
United Kingdom	1.5	200	1	50	400	200	100
Netherlands	1.25	200	1	50	400	200	100
France	3	180	2	60	600	300	120
Canada***	3	150	0.6	62	500	100	210
Sri Lanka	10	250	–	–	1000	400	–
Finland	1.5	100	1	100	1500	600	300
Spain*	3	200	2.5	100	1000	400	300
Austria	3–10	100–600	1–10	100–400	< 3000	< 700	100–600
USA	0.1–5	200–500	5	200	2000	800	600
Germany	10	900	8	200	2500	800	900
Ireland***	20	750	16	300	2500	1000	1000
Mexico**	0.7	45	0.4	0.25	200	70	70
Colombia	80	200	15	–	–	–	500
Brazil***	3.0	20	0.05	70	–	–	40
Digestate****	0.18–5	0.02–126	0.015–1.34	0.51–355.9	0.81–4019	1.4–681	0.06–560
World Health Organization	4	84	7	107	–	–	–
Food and Agriculture Organization	0.9–3	300	1	50	200	80	400
European Economic Commission	1–3	50–300	1–1.5	30–75	150–300	50–140	–

*In amendments (class C)

**As compost

***As fertilizers

****General characteristics for digestate from Food Waste

The use of digestate should always be incorporated into sound management and codes of practice and standards, as placing it without any prior treatment can end up damaging the soil rather than benefiting it. An example of this is phosphate overload, which can lead to diffuse pollution and excessive phosphorus concentrations (eutrophication) in coastal and inland waters, particularly in environmentally sensitive areas. This is evident in parts of Denmark, southwest Sweden, and Northern Ireland. In these areas, the recommended practice is to apply the digestate to meet the crop’s phosphorus needs and supplement nitrogen deficiencies with mineral fertilizer.

Many countries have established standards and policies for managing the risks associated with AD processes. The United Kingdom (BSI PAS 110: Producing Quality Anaerobic Digestate), Sweden (SPRC120), Germany (RAL GZ 245), Belgium (VLAREMA), Austria (ARGE), Switzerland (VKS-ASIC), Denmark (EC No. 834), and France (Standard NF U44-051), which outline specifications for physicochemical characteristics, system management, and contaminant concentration limits. In the United States, regulations governing digestate are covered under biosolids guidelines, with contaminant limits set by the USEPA (Lu et al. 2012). On the other hand, China allows the use of digestate as a feed supplement for various livestock and aquaculture species. However, national regulations restrict practice (Logan and Visvanathan 2019). While digestates have several benefits, many countries restrict their use or lack standardized regulations for their use. Peng and Pivato (2019) noted that the use of digestate as an agricultural product is permitted based on specific quality criteria, including the substrate of origin, the processes, and treatment techniques employed.

Digestate can be available in three forms: whole mixed, liquid, and solid. Each fraction can be applied to the soil as a destination once it meets the relevant regulatory standards and can be classified as a product (Nkoa 2014; Teglia et al. 2011). To comply with quality requirements, digestate must adhere to specific standards encompassing hygiene, impurities, degree of fermentation, odor, organic matter content, heavy metal concentration, and biological parameters (Al Seadi et al. 2013). Particularly concerning biological parameters, digestate used as fertilizer must ensure the absence of pathogens, viruses, and weed seeds.

As shown in Table 5, there is significant variability in permissible concentrations of heavy metals in soils, influenced by environmental policies and levels of regulation in each country. For example, Denmark enforces one of the world’s lowest Cd limits (0.8 mg/kg), whereas Colombia allows up to 80 mg/kg. Most digestates typically contain Cd concentrations ranging from 0.18 to 5 mg/kg, which may comply with standards in many regions but exceed the thresholds in more strictly regulated countries.

This variability underscores the importance of thorough digestate characterization, encompassing not only nutrient content but also contaminants such as heavy metals, prior to agricultural application. While many digestates meet the requirements in countries with more permissive regulations, they may pose risks in regions with stricter environmental standards. The lack of global regulatory consistency further complicates the development of unified guidelines. To ensure the safe and sustainable use of digestates, it is crucial to evaluate their composition in relation to both national and international regulatory frameworks, prioritizing soil integrity, plant health, and food safety.

### Digestates in Latin America

Between 2014 and 2018, a significant production deficit of fertilizers based on N, P_2_O_5_, and K_2_O occurred in Latin America and the Caribbean. The area faced a shortage of 525 million tons, 5193.4 million tons, and 5393 million tons per year, respectively. For instance, Brazil is one of the biggest consumers of fertilizers. In 2022, the total number of fertilizers delivered to the national market was 41 million tons, with 84% of this volume imported (Szychta et al. 2023). In 2021, Mexico produced 2.1 million tons of fertilizers. However, as in the case of Brazil, fertilizer demand is highly dependent on imports (4.8 million tons in 2019) related to nitrogenous fertilizers (61.5% of the total imports), followed by complex fertilizers (28.9%) and potassium fertilizers (6.2%) (Álvarez-González et al. 2023).

Farmers in Latin American countries often use digestate from low-tech digesters to fertilize agricultural land without proper quality testing or treatment. This increases the risks to human health, soil quality, and plant growth, including weed germination. A field study conducted by Garfí et al. (2011) showed that digestate from a manure-fed plastic tubular digester significantly increased potato and forage production. However, the study also highlighted the need for further research on the quality of digestate.

In Latin America, the use of digestates is a relatively recent development, which requires comprehensive studies to enhance our understanding of soil dynamics, crop suitability for different regions, and the characteristics of digestate content. Table 6 presents research conducted in Latin America that explores the application of digestates to enhance soil quality and agricultural crop productivity. However, a significant lack of quality standards for digestates and their applications. For example, studies in Mexico on winter triticale (Salcedo-Serrano et al. 2022) showed an increase (> 10%) in crop yield and seed efficiency compared with inorganic fertilizer. In contrast, bromatological evaluations and statistical analyses revealed that mineral uptake, as well as protein, sugar, and fiber content, were not significantly different (p > 0.05). Castro-Rivera et al. (2020) have shown promising results suggesting that digestate application enhances root growth in lettuce plants and improves germination rates. However, these studies failed to assess the heavy metal content, which highlights the need for further investigation before the widespread application of digestate in soil. Pathogen quantification was also conducted, revealing concentrations within permissible limits outlined by fertilizer and compost norms (Table 6).

**Table 6:** Examples of digestate research in Latin America

Country	Digestate’s substrate	Experiment	Results	Quality standard	References
Mexico	Bovine manure with tomato crop residues	Germination tests on lettuce seeds by placing the liquid part of the digestates	Digestate stimulated root elongation. The microbiological analysis of all the digestates complies with the permissible limits in the Environmental Technical Standard. It is not phytotoxic; an 80% dilution with water was added	NTA-006-SMA-RS-2006, for soil improvers	Castro-Rivera et al. (2020**)**
Peru	Bovine slurry	Digestate dilutions with water from 20 to 60%, added to onion cultures	50% digestate dilution presents the highest levels in improving crop yields	No use of any quality standards	Coaguila et al. (2019)
Colombia	Cattle manure and cheese whey	Use of digestate as (i) biofertilizer (sand filtered), (ii) compost (vermifilter), and (iii) directly applied to agricultural land without any post-treatment. Evaluation with the environmental impact with the Sima Pro 9.3 software	Compost was the most environmentally friendly scenario and increased the farmers income. The environmental impacts associated with post-treatment were considerably reduced (up to 9 times). Results are not considered for testing pathogens, toxicity, metals, or health effects	No use of any quality standards	Ziegler-Rodriguez et al. (2023)
Colombia	Fruit and vegetable waste (42%), pig and cow manure (42%), and grass (16%)	Physicochemical and microbiological analyses of the digestate	Digestate pH between 6.28 and a maximum of 7.26. Metals such as Cr, Nq, Hg, Pb, and Cd were lower than the Colombian standard referred to leachates. High values of phytopathogens (*Salmonella, Shiguella, Klebsiella, Pseudomonas,* and *Escherichia coli*) were recorded	Colombian regulations of to liquid discharges (Resolution 1074/1997)	Vega and Silva (2020)
Chile	Food waste	Evaluation of the use of digestate as an accelerator of wheat stubble degradation and later germination in radish seeds (*Raphanus sativus*)	Digestate favors the degradation of the wheat stubble, increasing the respiratory activity and total N content, accelerating the natural decomposition of the material, generating a stable product, without phytotoxic metabolites. The use of digestate raises the pH values from acidic to slightly alkaline. No phytotoxic effects were observed, with a germination index > 80% in all cases	Chilean standard of digestate quality 3375	Franchi (2016)
Brazil	Food waste	Germination tests on lettuce seeds (*Lactuca sativa*) with the liquid and solid part of the digestates in agricultural soils	The use of liquid and solid digestate increased the soil pH and improved the absorption of N and P, there was a greater increase in biomass in the lettuce plants in the 50% digestate treatment	Normative Instruction n° 61, Decreto 10375	Simon et al. (2023)

In some countries, such as Colombia, small-scale projects have been initiated by farmers or cooperative agencies to create inexpensive or low-tech biodigesters for waste treatment. These biodigesters produce biogas and digestate, which is often used as fertilizer without proper analysis or post-treatment. Researchers have recognized the potential risks associated with untreated digestate and explored methods to improve its quality for safe and effective use as fertilizer without harming the soil microbiota or crop health. A study conducted by Ziegler-Rodriguez et al. (2023) investigated two post-treatment techniques for digestates from cattle manure and whey: sand biofiltration and vermifiltration. The sand biofilter retained suspended solids and contaminants through a slow percolation process. In contrast, vermifiltration, which is based on vermicomposting, utilizes worms to decompose organic matter and effectively reduce heavy metal contamination. To assess the environmental impact, CO_2_ emissions were simulated, revealing that vermifiltration has a lower carbon footprint. This finding highlights its potential as a more sustainable treatment option.

Closed-loop technological systems have the potential to manage organic waste and generate revenue by producing fertilizer and biogas, thereby enhancing environmental sustainability. In Brazil, a comparison between AD plants using high solids AD process (VS > 20%) and wet AD showed that the former produced almost 2.5 times more solid digestate than the latter (233 kg/t OFMSW compared to 100 kg/t OFMSW in the Wet AD plant). The high solids AD process is also more robust, requires less maintenance, and has lower technical complexity, making it a suitable option for waste management, water conservation, energy systems, and biofertilizer production, considering the Brazilian context (Silva-Martínez et al. 2023). A successful case study on using a solid-state batch system for OFMSW treatment from the city of Rio de Janeiro has demonstrated not only the thermal generation (193 MWh_th_/month), but also a mass reduction of up to 40% of the initial digestate weight after thermal drying with effective in the hygienization of biosolids for agricultural purposes (600 kg of biosolids per ton of OFMSW), such as soil conditioning for recovery of a rainforest located within the City of Rio de Janeiro (Ornelas-Ferreira et al. 2020).

Further research is needed to investigate the quality and characteristics of digestate, particularly regarding its substrate and production process. While digestate has beneficial properties, it must meet quality standards in terms of pathogens, heavy metals, and antibiotics (Da Ros et al. 2018; Jiang et al. 2018). This necessitates the application of pretreatments to digestates, thereby increasing their quality to acceptable levels before application. Additionally, phytotoxicity or ecotoxicity analysis is necessary to assess the actual impact of the digestate on soil and crops (Da Ros et al. 2018). The variation in digestate composition has been identified as a bottleneck for its marketing, as even minor variations in substrates used in an AD process can lead to changes in digestate properties (Czekała et al. 2020). Therefore, digestate management and consumer demand depend on the digestate legal status as a by-product. Although some biofertilizers derived from AD products are already available in the market, different countries have varying regulations or even no specific legal framework for digestate use. In some cases, digestates are classified as waste, resulting in more expensive legal procedures for their recovery and marketing (Guilayn et al. 2019). However, if the AD process can be standardized to ensure the quality of the digestate and regulations are enforced to govern its use, it can be a valuable resource in promoting a circular economy for organic waste.

## Conclusion

Digestates are a promising alternative to traditional agricultural inputs, especially as organic fertilizers and soil enhancers. Their effectiveness largely depends on the composition of the original organic waste, which influences the nutrient content and microbial populations present. To ensure safe and practical application, it is crucial to evaluate both the quality of the digestate and the soil’s characteristics. Microbiological analyses have consistently demonstrated the presence of beneficial bacteria, including *Pseudomonas, Acinetobacter, Lactobacillus*, and Clostridiales. These microorganisms contribute to essential processes such as nutrient mineralization, phosphate solubilization, and the degradation of organic matter, all of which promote soil health and support plant growth. Additionally, digestates can act as prebiotics, stimulating microbial enzyme activities (e.g., dehydrogenase and β-glucosidase) and enhancing overall microbial biomass in soils.

Regarding heavy metals, studies show significant variations in the concentrations of Cd, Pb, Hg, Ni, Zn, Cu, and Cr in digestates. Although many concentrations meet the standards set by the FAO, WHO, and EEC, some untreated digestates exceed safe limits. This highlights the need for careful monitoring and treatment before agricultural application. In Latin America, the use of digestates is on the rise, driven by a dependence on imported fertilizers. Countries such as Mexico, Brazil, and Colombia have had promising experiences with digestates, but they face significant challenges, including a lack of clear regulations and limited assessments of health and environmental risks. Unregulated applications can pose significant hazards, underscoring the importance of establishing quality standards and effective post-treatment strategies.

## Data Availability

The data underlying this article will be shared on reasonable request to the corresponding author.
